# Synthesis of a Novel Pyrazine–Pyridone Biheteroaryl-Based Fluorescence Sensor and Detection of Endogenous Labile Zinc Ions in Lung Cancer Cells

**DOI:** 10.3390/s19092049

**Published:** 2019-05-02

**Authors:** Masayori Hagimori, Mana Taniura, Naoko Mizuyama, Yasushi Karimine, Shigeru Kawakami, Hideo Saji, Takahiro Mukai

**Affiliations:** 1 Department of Biophysical Chemistry, Kobe Pharmaceutical University, 4-19-1 Motoyamakita Machi, Higashinada Ku, Kobe 658-8558, Japan; mnnnm812@gmail.com; 2 Graduate School of Biomedical Sciences, Nagasaki University, 1-7-1 Sakamoto, Nagasaki 852-8501, Japan; bb30113014@ms.nagasaki-u.ac.jp (Y.K.); skawakam@nagasaki-u.ac.jp (S.K.); 3 Clinical Research Center, Nagasaki University Hospital, 1-7-1 Sakamoto, Nagasaki 852-8501, Japan; nhagimori@nagasaki-u.ac.jp; 4 Graduate School of Pharmaceutical Sciences, Kyoto University, 46–29 Yoshida-Shimoadachi-cho, Sakyo-ku, Kyoto 606-8501, Japan; hsaji@pharm.kyoto-u.ac.jp

**Keywords:** endogenous Zn^2+^, pyrazine–pyridone biheteroaryl, low background fluorescence, sulfonyl group, cellular imaging

## Abstract

A small extent of endogenous labile zinc is involved in many vital physiological roles in living systems. However, its detailed functions have not been fully elucidated. In this study, we developed a novel biheteroaryl-based low molecular weight fluorescent sensor, 3-(phenylsulfonyl)-pyrazine–pyridone (**5b**), and applied it for the detection of endogenous labile zinc ions from lung cancer cells during apoptosis. The electron-withdrawing property of the sulfonyl group between the phenyl ring as an electron donor and the pyridone ring as a fluorophore inhibited the intramolecular charge transfer state, and the background fluorescence of the sensor was decreased in aqueous media. From the structure–fluorescence relationship analysis of the substituent effects with/without Zn^2+^, compound **5b** acting as a sensor possessed favorable properties, including a longer emission wavelength, a large Stokes shift (over 100 nm), a large fluorescence enhancement in response to Zn^2+^ under physical conditions, and good cell membrane permeability in living cells. Fluorescence imaging studies of human lung adenocarcinoma cells (A549) undergoing apoptosis revealed that compound **5b** could detect endogenous labile zinc ions. These experiments suggested that the low molecular weight compound **5b** is a potential fluorescence sensor for Zn^2+^ toward understanding its functions in living systems.

## 1. Introduction

Essential trace elements are known to play important roles in many biochemical processes, including the construction of biological tissues and the sustaining and adjusting of physiological systems [1]. These elements show positive effects in physiological functions at optimal concentrations, whereas shortage or excessive concentrations affect biochemical processes and may evoke deficiency and toxicity. Because essential trace elements are both directly and indirectly related to diseases, detection with fluorescence imaging has become a useful tool in elucidating their distribution and dynamic states in living organisms, providing extensive information about diseases and facilitating the development of therapeutic strategies [2].

Zinc is the second-most abundant trace element in the living body and is involved in vital physiological roles such as enzyme regulation, gene expression, immune system response, and neurotransmission [2,3,4,5,6]. Most endogenous zinc is tightly bound to proteins for their structural and catalytic functions, whereas a small amount of zinc exists in free or labile forms in several tissues [6,7]. The disruption of zinc homeostasis causes serious damage related to neurodegenerative diseases, indicating that free and labile zinc plays an important role as an intracellular second messenger [7,8,9]. 

To detect and visualize the complicated physiological and pathological functions of zinc, various fluorescence Zn^2+^ sensors have been developed based on fluorophores such as coumarin, rhodamine, and cyanine [2,10,11,12]. These sensors have unique properties in selectivity and sensitivity. Recently, Zn^2+^ high-specific sensors discriminating Zn^2+^ from Cd^2+^ have been reported [13]. However, the details of zinc homeostatic processes are still unclear. Because the endogenous Zn^2+^ concentration is low (subnanomolar to millimolar range), high background fluorescence in a probe is a significant drawback in imaging small amounts of Zn^2+^ in living cells [12]. We previously reported pyridine–pyridone-based fluorescent Zn^2+^ sensors, in which the pyridine–pyridone core structure acted as both the chelating functionality for Zn^2+^ and the fluorescent moiety, such that the sensor has a low molecular weight (below 500 g mol^−1^) and has good cell membrane permeability [14,15,16]. The fluorescence mechanism of pyridine–pyridone sensors is the formation of an intramolecular charge transfer (ICT) state, and the separation between the phenyl ring as an electron-donor and the pyridone ring as a fluorophore influenced the push–pull system of the sensors, weakening the ICT and resulting in a decrease in background fluorescence [14]. The sensors could visualize the exogenously-added Zn^2+^ in living cells; however, we realized that further reducing the background fluorescence of the Zn^2+^ sensor was required to detect endogenous Zn^2+^.

Herein, we reported novel biheteroaryl Zn^2+^ fluorescence compounds (**3a,b**, **4a,b**, and **5a,b**), which have a sulfonyl group as a strong electron-withdrawing group inhibiting the push–pull system, thus strongly reducing the background fluorescence of the sensors. The introduction of substituents also affected fluorescence properties such as intensity, excitation, and emission wavelength with or without Zn^2+^. From the structure–fluorescence relationship analysis of the substituent effects in the pyridone core structure, 3-(phenylsulfonyl)-pyrazine–pyridone (**5b**), with pyrazine and tosyl groups, exhibited favorable properties as a Zn^2+^ sensor. In this paper, we described the design and synthesis of fluorescence compounds and tested a biological application that successfully indicated that sensor **5b** could detect endogenous Zn^2+^ in lung cancer cells.

## 2. Materials and Methods

### 2.1. Materials and Instruments

All chemicals were of the highest purity available. ^1^H-NMR was measured on a Varian Mercury-300 (300 MHz) with chemical shifts reported as ppm. Mass spectra (MS) and high-resolution MS (HRMS) were recorded on a Thermo Fisher Scientific Exactive spectrometer. Fluorescence spectra were obtained on a F7000 fluorescence spectrophotometer (Hitachi High-Tech, Tokyo, Japan) and a RF-5300PC fluorescence spectrophotometer (Shimadzu, Kyoto, Japan). 

### 2.2. Synthesis of 4-(Methylsulfanyl)-5-(Phenylsulfonyl)-[2,2’-Bipyridin]-6(1H)-One (**3a**)

3,3-Bis(methylsulfanyl)-2-(phenylsulfonyl)acrylonitrile (**2a**; 2.84 g, 10 mmol) [17,18] was slowly added to a solution of 2-acetylpyridine (**1a**; 1.21 g, 10.0 mmol) and sodium hydroxide (0.56 g, 14 mmol) in DMSO over 30 min, and the mixture was stirred for 2 h at room temperature. After adding sodium hydroxide (0.56 g, 14 mmol), the mixture was stirred for an additional 3 h at room temperature. The reaction mixture was poured into 100 mL of water and adjusted to pH 3–4 with a 10% hydrochloric acid solution. The mixture was extracted with 100 mL of chloroform three times. Organic extracts were combined, washed with water, and dried over anhydrous sodium sulfate. After concentration in vacuo, the residue was refluxed in 1% hydrochloric acid solution for 1 h. The precipitate that appeared was collected by filtration, washed with water, and recrystallized from methanol to give **3a** (0.27 g, 5.7 mmol, 7.5%) as brown crystals. Melting point (Mp) 331–332 °C. ^1^H-NMR (DMSO-d_6_, 300 MHz): δ 2.71 (s, 3H), 7.01 (s, 1H), 7.55–7.64 (m, 3H), 7.90–7.98 (m, 3H), 8.18 (d, *J* = 8.4 Hz, 2H), 8.74 (d, *J* = 4.5 Hz, 1H), 11.76 (brs, 1H). MS m/z: 359 [M + H^+^]. HRMS calcd. for C_17_H_15_N_2_O_3_S_2_ [M + H^+^]: 359.0524. Found: 359.0518.

### 2.3. Synthesis of 4-(Methylsulfanyl)-5-Tosyl-[2,2’-Bipyridin]-6(1H)-One (**3b**)

Compound **3b** (0.67 g, 1.8 mmol) was prepared in 39% yield from 0.60 g (5.0 mmol) of **1a** and 1.5 g (5.0 mmol) of 3,3-bis(methylsulfanyl)-2-tosylacrylonitrile (**2b**) in a manner similar to that described for the synthesis of **3a**. An analytical sample was recrystallized from methanol to give dark gray crystals. Mp > 400 °C. ^1^H-NMR (DMSO-d_6_, 300 MHz): δ 2.41 (s, 3H), 2.71 (s, 3H), 6.98 (s, 1H), 7.46 (d, *J* = 8.4 Hz, 2H), 7.54–7.58 (m, 3H), 7.77 (d, *J* = 8.4 Hz, 2H), 8.71 (d, *J* = 3.9 Hz, 1H). MS m/z: 371 [M – H^+^]. HRMS calcd. for C_18_H_15_N_2_O_3_S_2_ [M – H^+^]: 371.0524. Found: 371.0532.

### 2.4. Synthesis of 4’-Methyl-4-(Methylsulfanyl)-5-(Phenylsulfonyl)-[2,2’-Bipyridin]-6(1H)-One (**4a**)

Compound **4a** (1.14 g, 3.0 mmol) was prepared in 61% yield from 0.68 g (5.0 mmol) of 2-acetyl-4-methylpyridine (**1b**) and 1.42 g (5.0 mmol) of **2a** in a manner similar to that described for the synthesis of **3a**. An analytical sample was recrystallized from methanol to give dark gray crystals. Mp 221–222 °C. ^1^H-NMR (DMSO-d_6_, 300 MHz): δ 2.51 (s, 3H), 2.70 (s, 3H), 6.91 (s, 1H), 7.25 (d, *J* = 4.8 Hz, 1H), 7.48 (s, 1H), 7.55–7.62 (m, 3H), 7.75 (d, *J* = 7.8 Hz, 2H), 8.59 (d, *J* = 4.8 Hz, 1H). MS m/z: 371 [M – H^+^]. HRMS calcd. for C_18_H_15_N_2_O_3_S_2_ [M – H^+^]: 371.0524. Found: 371.0531.

### 2.5. Synthesis of 4’-Methyl-4-(Methylsulfanyl)-5-Tosyl-[2,2’-Bipyridin]-6(1H)-One (**4b**)

Compound **4b** (0.31 g, 0.8 mmol) was prepared in 27% yield from 0.41 g (3.0 mmol) of **1b** and 0.89 g (3.0 mmol) of **2b** in a manner similar to that described for the synthesis of **3a**. An analytical sample was recrystallized from methanol to give dark gray crystals. Mp > 400 °C. ^1^H-NMR (DMSO-d_6_, 300 MHz): δ 2.39 (s, 3H), 2.52 (s, 3H), 2.70 (s, 3H), 6.91 (s, 1H), 7.34–7.45 (m, 4H), 7.62 (d, *J* = 8.4 Hz, 2H), 8.59 (d, *J* = 4.2 Hz, 1H). MS m/z: 385 [M – H^+^]. HRMS calcd. for C_19_H_17_N_2_O_3_S_2_ [M – H^+^]: 385.0681. Found: 385.0689.

### 2.6. Synthesis of 4-(Methylsulfanyl)-3-(Phenylsulfonyl)-6-(Pyrazin-2-Yl)Pyridin-2(1H)-One (**5a**)

Compound **5a** (0.16 g, 0.5 mmol) was prepared in 9% yield from 0.61 g (5.0 mmol) of 2-acetylpyrazine (**1c**) and 1.42 g (5.0 mmol) of **2a** in a manner similar to that described for the synthesis of **3a**. An analytical sample was recrystallized from methanol to give dark gray crystals. Mp 188–189 °C. ^1^H-NMR (DMSO-d_6_, 300 MHz): δ 2.73 (s, 3H), 7.09 (s, 1H), 7.60 (m, 2H), 7.66 (m, 1H), 7.70 (d, *J* = 7.2 Hz, 2H), 8.76 (d, *J* = 1.8 Hz, 1H), 8.83 (d, *J* = 1.5 Hz, 1H), 9.12 (s, 1H). MS m/z: 358 [M – H^+^]. HRMS calcd. for C_16_H_12_N_3_O_3_S_2_ [M – H^+^]: 358.0320. Found: 358.0331.

### 2.7. Synthesis of 4-(Methylsulfanyl)-6-(Pyrazin-2-Yl)-3-Tosylpyridin-2(1H)-One (**5b**)

Compound **5b** (0.19 g, 0.5 mmol) was prepared in 10% yield from 0.61 g (5.0 mmol) of **1c** and 1.50 g (5.0 mmol) of **2b** in a manner similar to that described for the synthesis of **3a**. An analytical sample was recrystallized from methanol to give dark gray crystals. Mp 232–233 °C. ^1^H-NMR (DMSO-d_6_, 300 MHz): δ 2.38 (s, 3H), 2.72 (s, 3H), 7.13 (s, 1H), 7.41 (d, *J* = 8.1 Hz, 2H), 7.82 (d, *J* = 8.4 Hz, 2H), 8.80 (d, *J* = 0.9 Hz, 1H), 8.83 (d, *J* = 1.5 Hz, 1H), 9.12 (s, 1H). MS m/z: 372 [M – H^+^]. HRMS calcd. for C_17_H_14_N_3_O_2_S_2_ [M – H^+^]: 372.0477. Found: 372.0484.

### 2.8. Spectral Measurement Studies

A stock solution of each compound (1 mM) was prepared by dissolving in DMSO. Solutions of perchlorate salts of metal ions (Al^3+^, Ca^2+^, Cd^2+^, Co^2+^, Cu^2+^, Fe^2+^, Fe^3+^, K^+^, Mg^2+^, Mn^2+^, Na^+^, Pb^2+^, and Zn^2+^) were prepared by dissolving in distilled water. The fluorescence of test compounds (10^−5^ M) in HEPES buffer (100 mM, 5% DMSO, pH = 7.4) were analyzed in the presence of Zn^2+^. Job’s plot was used to investigate the binding stoichiometries of **3a,b**, **4a,b**, and **5a,b** to Zn^2+^. The dissociation constant (*K*_d_) values were investigated by the following Benesi–Hildebrand plot [19,20].

1/(*F* – *F*_0_) = 1/{*K*_a_(*F_max_* – *F*_0_)[Zn^2+^]^n^} + 1/(*F_max_* – *F*_0_)

where *F* is the fluorescence intensity, *F*_0_ is the fluorescence intensity without Zn^2+^, and *F_max_* is the fluorescence in addition of excess Zn^2+^. The association constant (*K*_a_) is the inverse of *K*_d_ and is determined from the slope of the straight line of the plot of 1/(*F* – *F*_0_) against 1/[Zn^2+^]. The selectivity of each compound was investigated in HEPES buffer (100 mM, 5% DMSO, pH = 7.4). The fluorescence quantum yields were measured with respect to a quinine sulfate solution (Φ = 0.54) as the standard.

### 2.9. Cellular Imaging by Fluorescence Microscope

A549 human lung cancer cells were cultured in Dulbecco’s modified Eagle’s medium (DMEM) that included 10% fetal bovine serum and 1% penicillin at 37 °C in a humidified atmosphere with 5% CO_2_. To investigate the cell-membrane permeability of **5b**, the cells were incubated with 100 μM of 1:1 Zn^2+^/pyrithione in the culture media for 30 min at 37 °C. After washing with phosphate-buffered saline (PBS), the treated cells were incubated with **5b** (100 μM) in the culture media for 30 min at 37 °C. The incubated cells were imaged with fluorescence microscopy (Nikon Eclipse Ti). For *N*,*N*,*N’*,*N’*-tetrakis(2-pyridylmethyl)ethylenediamine (TPEN) studies, the cells were incubated with 1:1 Zn^2+^/pyrithione (100 μM) in the culture media for 30 min at 37 °C and washed with PBS. After incubating with **5b** (100 μM) in the culture media for 30 min at 37 °C and washing with PBS, the cells were incubated with 100 μM of TPEN for an additional 30 min at 37 °C and imaged with fluorescence microscopy (Nikon Eclipse Ti).

To detect endogenous Zn^2+^, the cells were incubated with hydrogen peroxide (200 μM) in DMEM for 24 h at 37 °C. After washing with PBS, the cells were incubated with **5b** (100 μM) in the culture media for 30 min at 37 °C. The incubated cells were imaged with fluorescence microscopy (Nikon Eclipse Ti). For TPEN studies, the cells were incubated with hydrogen peroxide (H_2_O_2_, 200 μM) in the culture media for 24 h at 37 °C and washed with PBS. After incubation with **5b** (100 μM) in culture media for 30 min at 37 °C and washing with PBS, the cells were incubated with 100 μM of TPEN for an additional 30 min at 37 °C and imaged with fluorescence microscopy (Nikon Eclipse Ti).

## 3. Results

**Figure 1:**
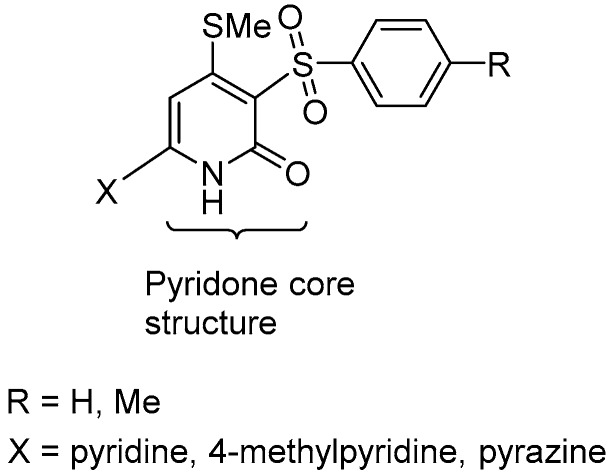
Schematic structure of novel fluorescence Zn^2+^ sensors.

**Scheme 1:**
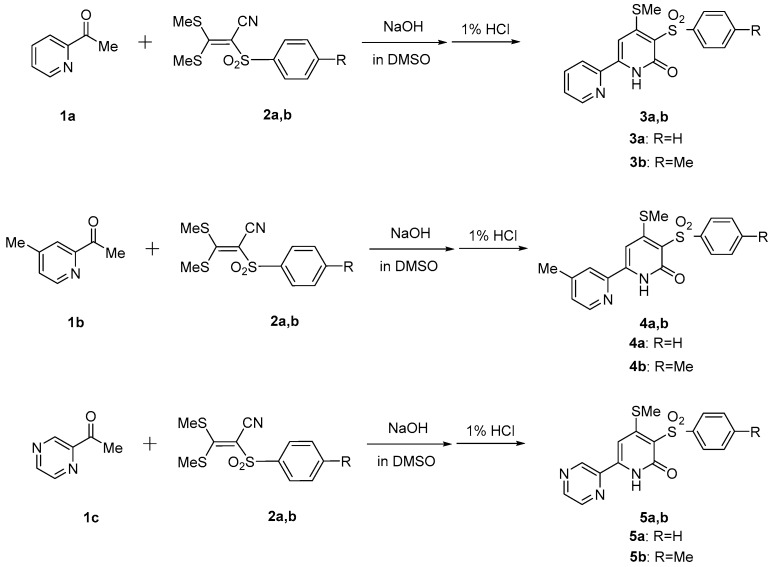
Synthesis of compounds **3a,b**, **4a,b,** and **5a,b**.

Our previous research demonstrated that the NH/OH proton of the pyridone core structure acted as a fluorescence off–on switch and that the weakening of the electron transfer from the electron-donating group to the pyridone core structure reduced the background fluorescence of the Zn^2+^ sensors [14,15]. Then, to develop a lower background fluorescence sensor, we introduced benzensulfonyl or tosyl groups at the two-position of the pyridone ring (Figure 1). In addition, Zn^2+^ was bound to the bipyridyl form. Therefore, we introduced pyridine, 4-methylpyridine, or pyrazine as an electron-withdrawing heteroaryl group at the six-position of the pyridone ring. Pyrazine is a nitrogen-containing, six-membered heterocycle, which has lower basicity and a higher π-acceptor ability than pyridine [21]. Therefore, it was expected that the replacement of pyridine with pyrazine would affect the compound’s coordination ability and fluorescent property towards Zn^2+^. Compound **3a** was synthesized from a one-pot reaction of 2-acetylpyridine (**1a**) with sulfonyl ketene dithioacetal (**2a**) in the presence of NaOH as a base, followed by treatment with 1% HCl (Scheme 1). In a similar manner, compound **3b**, having a tosyl group on the two-position of the pyridone ring, was obtained in 39% yield. Compounds **4a** and **4b**, having a 4-methyl-2-pyridyl group, were prepared from the reaction of 2-acetyl-4-methylpyridine (**1b**) and sulfonyl ketene dithioacetal (**2a,b**). The reaction of 2-acetylpyrazine (**1c**) with sulfonyl ketene dithioacetals (**2a,b**) afforded pyrazine–pyridone compounds (**5a,b**) in 9% and 10% yields, respectively.

**Figure 2:**
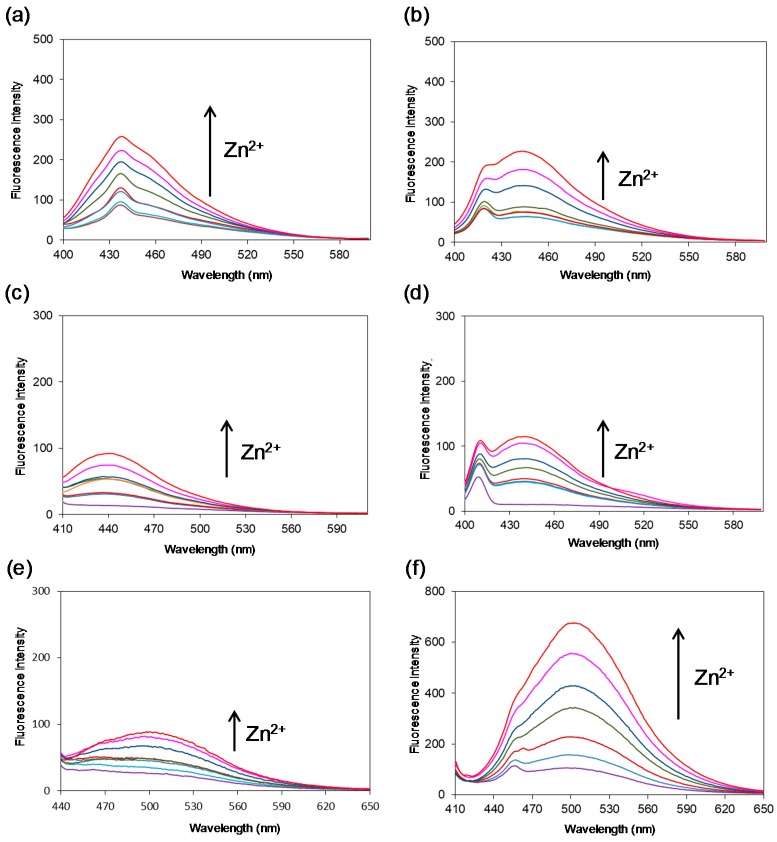
Fluorescence spectra of (**a**) **3a** (10^−5^ M, λ_ex_ = 381 nm); (**b**) **3b** (10^−5^ M, λ_ex_ = 367 nm); (**c**) **4a** (10^−5^ M, λ_ex_ = 353 nm); (**d**) **4b** (10^−5^ M, λ_ex_ = 360 nm); (**e**) **5a** (10^−5^ M, λ_ex_ = 377 nm); (**f**) **5b** (10^−5^ M, λ_ex_ = 395 nm) upon addition of Zn^2+^ in the form of perchlorate salt (0, 1, 2.5, 5, 10, 50, 100 μM) in HEPES buffer (100 mM, 5% DMSO, pH = 7.4). λ_ex_ = excitation wavelength. The narrow fluorescence band of (**a**), (**b**), (**d**), and (**e**) were the Raman scattering peak of the solvent.

**Figure 3:**
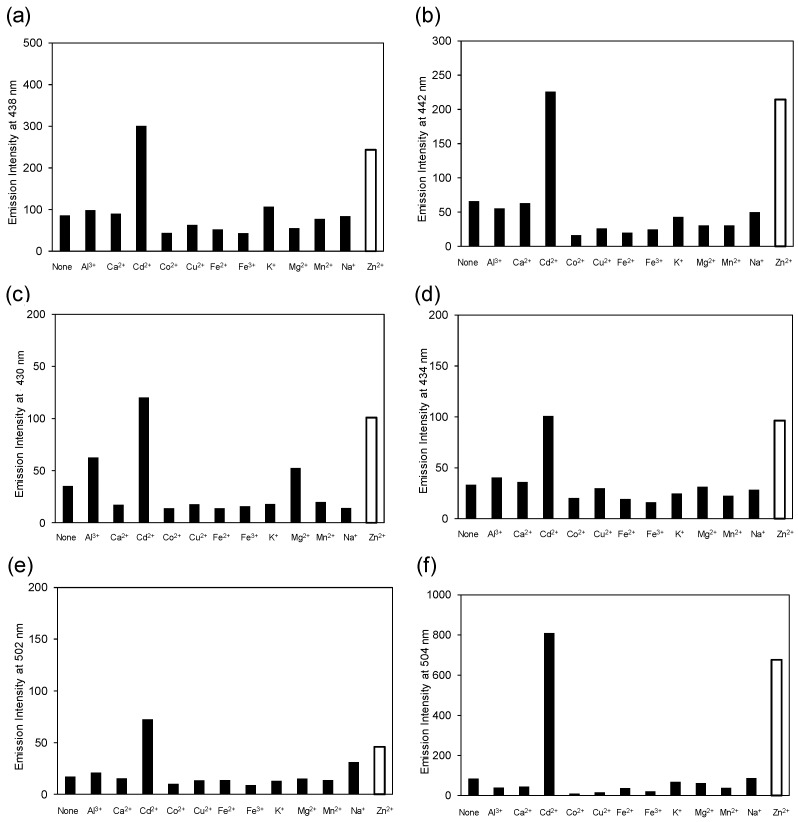
Fluorescence responses of (**a**) **3a** (10^−5^ M, λ_em_ = 381 nm); (**b**) **3b** (10^−5^ M, λ_em_ = 367 nm); (**c**) **4a** (10^−5^ M, λ_em_ = 353 nm); (**d**) **4b** (10^−5^ M, λ_em_ = 360 nm); (**e**) **5a** (10^−5^ M, λ_em_ = 377 nm); (**f**) **5b** (10^−5^ M, λ_em_ = 395 nm) upon addition of different metal cations in HEPES buffer (100 mM, 5% DMSO, pH = 7.4). λ_em_ = emission wavelength.

Compounds **3a,b**, **4a,b**, and **5a,b** were soluble in aqueous media after dilution of their DMSO stock solutions, and all spectroscopic measurements were performed in HEPES buffer (100 mM, 5% DMSO, pH 7.4). Figure 2 and Table 1 show the fluorescence spectra and corresponding data of **3a,b**, **4a,b**, and **5a,b**. All compounds were excited by 353–395 nm light, and the background fluorescence quantum yields (Φ) of the compounds were in the range of 4.1 × 10^−4^–9.8 × 10^−4^, which were considerably lower than previously reported pyridine–pyridone type compounds without a sulfonyl group [14,15,16]. As expected, these results suggested that introduction of a sulfonyl group between the pyridone core structure inhibits electron transfer from the electron-donating group and reduces the background fluorescence of **3a,b**, **4a,b**, and **5a,b**. After the addition of Zn^2+^, the fluorescence intensities of all compounds gradually increased in a concentration-dependent manner with Zn^2+^. Compounds **3a,b** and **4a,b** exhibited blue fluorescence at 430–442 nm, and the emission maximum wavelengths of **3b** and **4b** exhibited 4–5 nm bathochromic shifts by the introduction of a methyl group at the para position of the phenyl ring. In contrast, the methyl group of the 4-methyl-2-pyrigyl ring of **4a,b** induced 7–8 nm hypsochromic shifts in the emission maximum wavelengths. In pyrazine compounds **5a,b**, small bathochromic shifts were observed by introduction of the methyl group at the para position of the phenyl ring. On the other hand, the emission maximum wavelengths of compounds **5a,b** showed large bathochromic shifts, approximately 60–70 nm, compared to compounds **3a,b** and **4a,b** with pyridyl moieties, and **5a,b** emitted blue–green fluorescence around 500 nm. Since the emission maximum wavelengths of all previously reported pyridine–pyridone-type compounds were near 400–450 nm [14,15,16], it is noteworthy that the emission wavelength could be tuned by the simple replacement of pyridine with pyrazine. In addition, compounds **5a,b** showed very large Stokes shifts (over 100 nm), which became an advantage for detecting Zn^2+^ without too much interference from the excitation light. The binding analysis of compounds **3a,b**, **4a,b**, and **5a,b** and Zn^2+^ indicated that the complex formations had 1:1 stoichiometries (Supplementary data). We also calculated the dissociation constants (*K*_d_) of **3a,b**, **4a,b**, and **5a,b** from the Benesi–Hildebrand equation [18,19] using fluorescent titration data (Supplementary data). The *K*_d_ values of **5a** and **5b** were 59 × 10^−5^ M and 32 × 10^−5^ M, respectively, which were higher than those of **3a,b** and **4a,b** (1.6 × 10^−5^–9.4 × 10^−5^ M). These results indicated that the replacement of pyridine with pyrazine obviously influenced the compound’s coordination ability and fluorescent property with Zn^2+^. To investigate the selectivity of these compounds, various cations including Al^3+^, Ca^2+^, Cd^2+^, Co^2+^, Cu^2+^, Fe^2+^, Fe^3+^, K^+^, Mg^2+^, Mn^2+^, Na^+^, and Zn^2+^ were added to solutions of **3a,b**, **4a,b**, and **5a,b** in HEPES buffer (100 mM, 5% DMSO, pH = 7.4). As shown in Figure 3, all compounds showed selectivity toward Zn^2+^, and especially the pyrazine compound **5b** exhibited a large chelation-enhanced fluorescence (CHEF) effect with Zn^2+^ and exhibited an 8.1-fold enhancement over **5b** without Zn^2+^ addition. Fluorescence enhancement was also observed upon addition of Cd^2+^. Zn^2+^ and Cd^2+^ belong to the same group of the periodic table, therefore it has been reported that Cd^2+^ interfered with the detection of Zn^2+^ in various types of Zn^2+^ sensors [14,15,16,22,23]. However, Cd^2+^ is not naturally occurring in living systems, so it may have little influence on visualizing cellular Zn^2+^. With addition of other metal ions (Al^3+^, Ca^2+^, Co^2+^, Cu^2+^, Fe^2+^, Fe^3+^, K^+^, Mg^2+^, Mn^2+^, and Na^+^), no large fluorescence changes, including both CHEF effects and chelation-enhanced fluorescence quenching (CHEQ) effects, were observed. We also evaluated the fluorescence change with Pb^2+^ having a wide-spectrum toxic effect in living systems, however both CHEF and CHEQ effects were not observed (Supplementary data).

Among the compounds **3a,b**, **4a,b**, and **5a,b**, pyrazine compound **5b** had the most useful properties, namely low background fluorescence, emitted fluorescence at 504 nm, a large Stokes shift (over 100 nm), and an 8.1-fold fluorescence enhancement with Zn^2+^. Next, we performed further experiments, including a competition experiment, a test of pH influence, and cellular fluorescence imaging, to demonstrate the sensing ability of **5b**.

**Figure 4:**
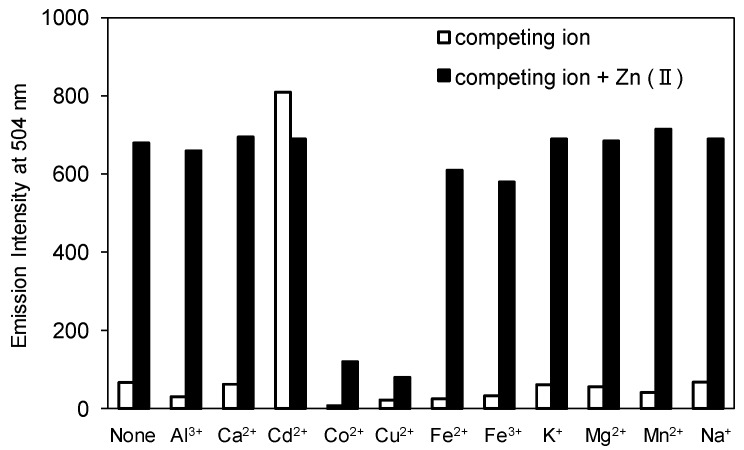
Competitive binding experiments of **5b**, in which competing metal ions (10^−3^ M) and Zn^2+^ (10^−3^ M) were added to **5b** (10^−5^ M) in HEPES buffer (100 mM, 5% DMSO, pH = 7.4).

**Figure 5:**
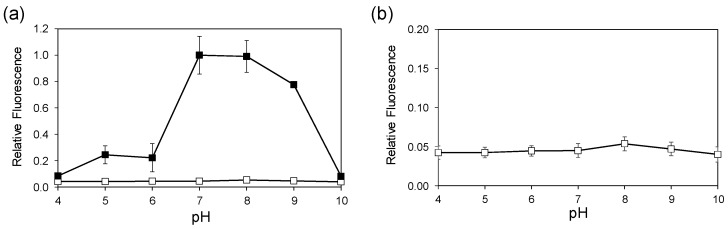
(**a**) Effect of pH on the fluorescence properties of **5b** in the absence (□) and presence (■) of Zn^2+^. (**b**) Enlarged figure of **5b** in the absence (□) of Zn^2+^.

Figure 4 shows the results of the competition experiment of **5b** between Zn^2+^ and other metal ions. The CHEF effect with Zn^2+^ was quenched by the addition of Co^2+^ or Cu^2+^, whereas it was not affected by addition of alkali-metal ions (Na^+^ and K^+^) or group 2 ions (Ca^2+^ and Mg^2+^), which exist abundantly in millimolar concentrations in the living body. The fluorescence intensity with Cd^2+^ was slightly weakened by the addition of Zn^2+^. Next, we evaluated the fluorescence changes of **5b** from pH 4.0 to 10.0 in the absence and presence of Zn^2+^ (Figure 5). The fluorescence intensity of **5b** with Zn^2+^ decreased in acidic conditions (pH 4.0 to 6.0). Protons might reflect the complexation Zn^2+^ with **5b**. On the other hand, the fluorescence intensity of the emission maximum at 504 nm was stable within the pH range 7.0–8.0. These results indicated that compound **5b** could be utilized for cellular experiments under physiological conditions. 

**Figure 6:**
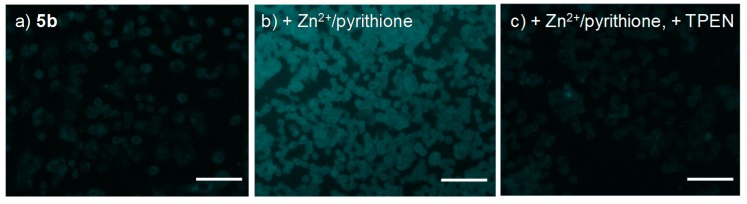
Fluorescence images of exogenously added Zn^2+^ in A549 cells: (**a**) Fluorescence image of cells with **5b** (100 μM); (**b**) fluorescence image incubated with **5b** (100 μM) in the presence of Zn^2+^/pyrithione (100 μM); (**c**) fluorescence image of (**b**) after treatment with TPEN (100 μM). Scale bars = 300 μm.

**Figure 7:**
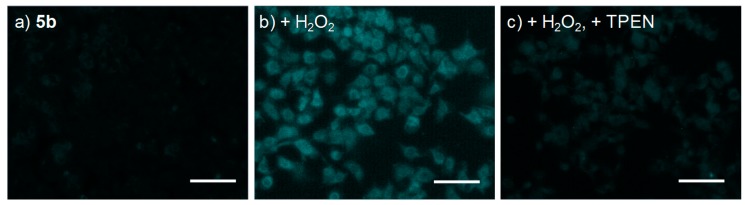
Fluorescence images of endogenous Zn^2+^ in A549 cells: (**a**) Fluorescence image of cells with **5b** (100 μM); (**b**) fluorescence image incubated with **5b** (100 μM) after incubation with H_2_O_2_ (200 μM) for 24 h; (**c**) fluorescence image of (**b**) after treatment with TPEN (100 μM). Scale bars = 300 μm.

To evaluate the efficacy of **5b** for living cell Zn^2+^ imaging, we conducted fluorescence microscopy studies in human lung adenocarcinoma cells (A549). The cells incubated with **5b** showed a negligible fluorescence due to its low background fluorescence (Figure 6a). After addition of both **5b** (100 μM) and Zn^2+^/pyrithione (100 μM), bright intracellular fluorescence was observed (Figure 6b). The addition of TPEN as a Zn^2+^ high-affinity chelator decreased the cellular fluorescence of **5b** (Figure 6c) [24]. These results indicated that compound **5b** possessed good cell membrane permeability and that the intracellular fluorescence change was due to the selective interaction between compound **5b** and Zn^2+^. In these experiments, zinc toxicity was not observed. We further evaluated the ability of **5b** to detect endogenous Zn^2+^ in apoptotic cells. It has been reported that Zn^2+^ is released from intracellular zinc stores when the cells are in the apoptosis stage [25]. Fluorescence enhancement was not observed in A549 cells after the induction of apoptosis by incubation with H_2_O_2_ (200 μM) for 24 h (Figure 7a). On the other hand, bright fluorescence was observed when compound **5b** was supplied to the cells and incubated for 30 min at 37 °C (Figure 7b). To investigate fluorescence enhancement due to the interaction between compound **5b** and endogenous Zn^2+^, the cells treated with H_2_O_2_ for 24 h were additionally incubated with TPEN after treatment with **5b**. As shown in Figure 7c, the intracellular fluorescence decreased upon the addition of TPEN, suggesting that compound **5b** could detect endogenous labile zinc ions. 

## 4. Conclusions

To detect endogenous Zn^2+^, we designed and synthesized low-background-fluorescence sensors **3a,b**,**4a,b**, and **5a,b** from one-pot reactions of 2-acetylpyridines, 2-acetyl-4-methylpyridine, or 2-acetylpyrazine with sulfonyl ketene dithioacetals and investigated their fluorescence properties. The sulfonyl group between the phenyl ring and the three-position of the pyridone ring affected the ICT state, and the background fluorescence of **3a,b**,**4a,b**, and **5a,b** was considerably decreased. The CHEF effects of **3a,b**,**4a,b**, and **5a,b** were observed upon addition of Zn^2+^. Methyl substitution on the R positions of **3a**, **4a**, and **5a** influenced the emission maximum wavelengths, and the replacement of pyridine with pyrazine induced large, approximately 60–70 nm bathochromic shifts. Upon addition of Zn^2+^, the pyrazine compound **5b** exhibited favorable properties, including an emission wavelength at 504 nm, a large Stokes shift (over 100 nm), a large fluorescence enhancement, Zn^2+^ selectivity, and stability in physiological pH conditions. Furthermore, compound **5b** exhibited favorable cell membrane permeability and selective detection of Zn^2+^ in living human lung adenocarcinoma A549 cells and visualized endogenous labile zinc ions from the cells during apoptosis. We expect that the pyrazine–pyridone biheteroaryl-based compound **5b** will contribute toward a better understanding of Zn^2+^ biological functions. 

## Figures and Tables

**Table 1:** Fluorescence data for compounds **3a,b**, **4a,b**, and **5a,b**.

Compounds	E_x_ max (nm)	E_m_ max (nm)	SS^a^ (nm)	Ф^b^ Zn^2+^ free (×10^–4^)	Ф^b^ Zn^2+^ complex (×10^−4^)	*K*_d_ (×10^−5^ M)
**3a**	381	438	57	9.8 ± 3.0	35 ± 3	8.0 ± 7.1
**3b**	367	442	75	9.2 ± 0.9	21 ± 7	5.5 ± 3.3
**4a**	353	430	77	4.1 ± 1.0	10 ± 4	9.4 ± 3.4
**4b**	360	435	75	5.1 ± 1.7	11 ± 1	1.6 ± 1.4
**5a**	377	502	125	4.5 ± 1.1	10 ± 4	59 ± 24
**5b**	395	504	109	5.4 ± 1.6	40 ± 13	32 ± 25

^a^ Stokes shift. ^b^ The fluorescence quantum yield values were measured with respect to quinine sulfate solution as standard.

## Supplementary Materials

The following are available online at https://www.mdpi.com/1424-8220/19/9/2049/s1, Figure S1: Job’s plot analysis of (a) **3a** (10^−5^ M, λ_em_ = 381 nm); ); (**b**) **3b** (10^−5^ M, λ_em_ = 367 nm); (**c**) **4a** (10^−5^ M, λ_em_ = 353 nm); (**d**) **4b** (10^−5^ M, λ_em_ = 360 nm); (**e**) **5a** (10^−5^ M, λ_em_ = 377 nm); (**f**) **5b** (10^−5^ M, λ_em_ = 395 nm). The total concentration of each compound and Zn^2+^ are 10 μM in HEPES buffer (100 mM, 5% DMSO, pH = 7.4). λ_em_ = emission wavelength. Figure S2: Typical Benesi-Hildebrand analysis of (**a**) **3a**, (**b**) **3b**, (**c**) **4a**, (**d**) **4b**, (**e**) **5a** and (**f**) **5b**. Figure S3: Fluorescence responses of (**a**) **3a** (10^−5^ M, λ_em_ = 381 nm); (**b**) **3b** (10^−5^ M, λ_em_ = 367 nm); (**c**) **4a** (10^−5^ M, λ_em_ = 353 nm); (**d**) **4b** (10^−5^ M, λ_em_ = 360 nm); (**e**) **5a** (10^−5^ M, λ_em_ = 377 nm); (**f**) **5b** (10^−5^ M, λ_em_ = 395 nm) upon addition of Pb^2+^ in HEPES buffer (100 mM, 5% DMSO, pH = 7.4). λ_em_ = emission wavelength.
