# Supplementary material for: Synthesis of a Novel Pyrazine–Pyridone Biheteroaryl-Based Fluorescence Sensor and Detection of Endogenous Labile Zinc Ions in Lung Cancer Cells
Source: Sensors (Basel). 2019 May 2;19(9):2049. doi: 10.3390/s19092049 (PMC6540122; doi:10.3390/s19092049)
Supplement: Supplementary file 1 [file sensors-19-02049-s001.pdf]

Supplementary Materials

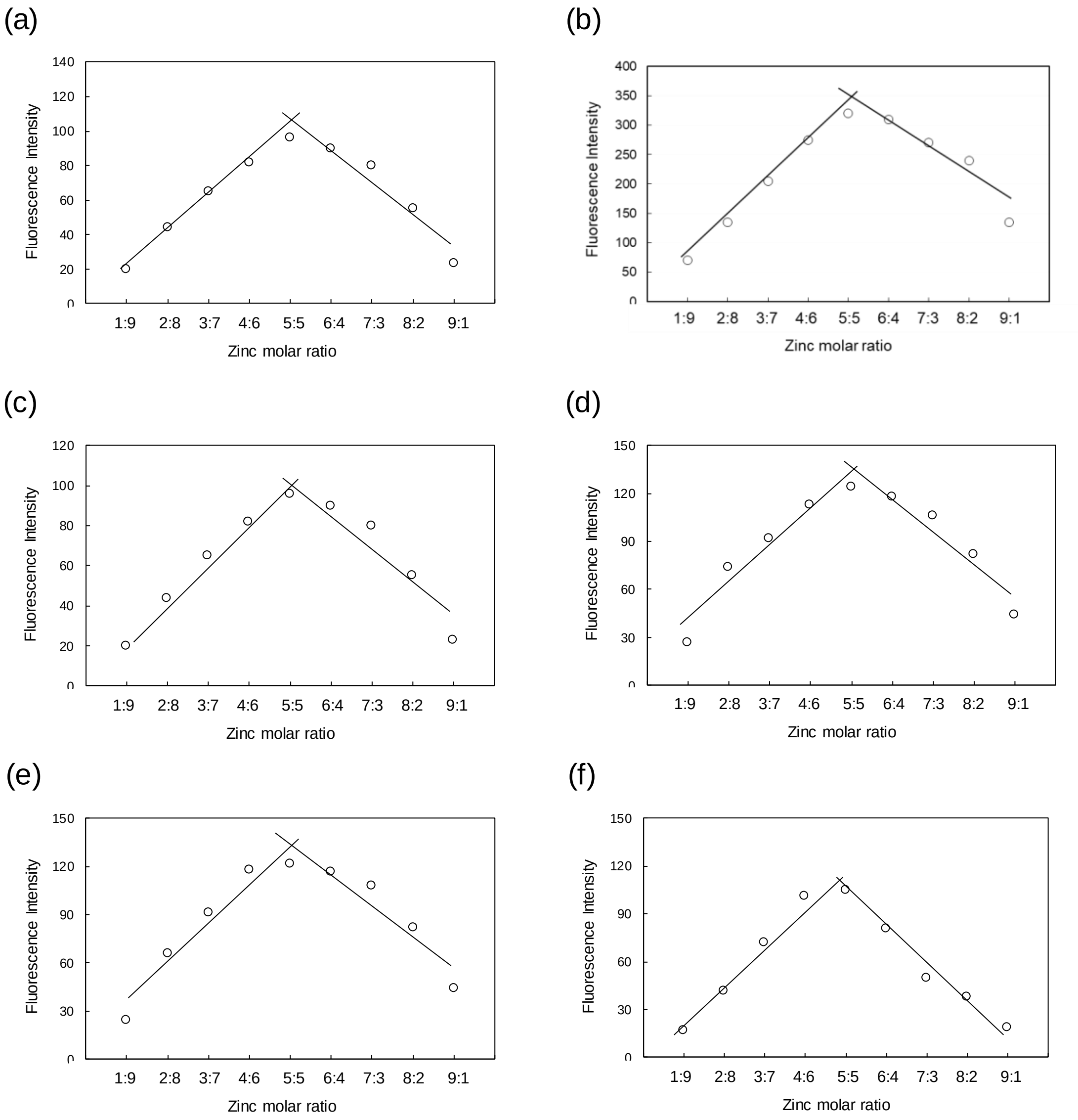

Figure S1. Job's plot analysis of (a) **3a** (10^{-5} M, λ_{em} = 381 nm); (b) **3b** (10^{-5} M, λ_{em} = 367 nm); (c) **4a** (10^{-5} M, λ_{em} = 353 nm); (d) **4b** (10^{-5} M, λ_{em} = 360 nm); (e) **5a** (10^{-5} M, λ_{em} = 377 nm); (f) **5b** (10^{-5} M, λ_{em} = 395 nm). The total concentration of each compound and Zn2+ are 10 μM in HEPES buffer (100 mM, 5% DMSO, pH = 7.4). λ_{em} = emission wavelength.

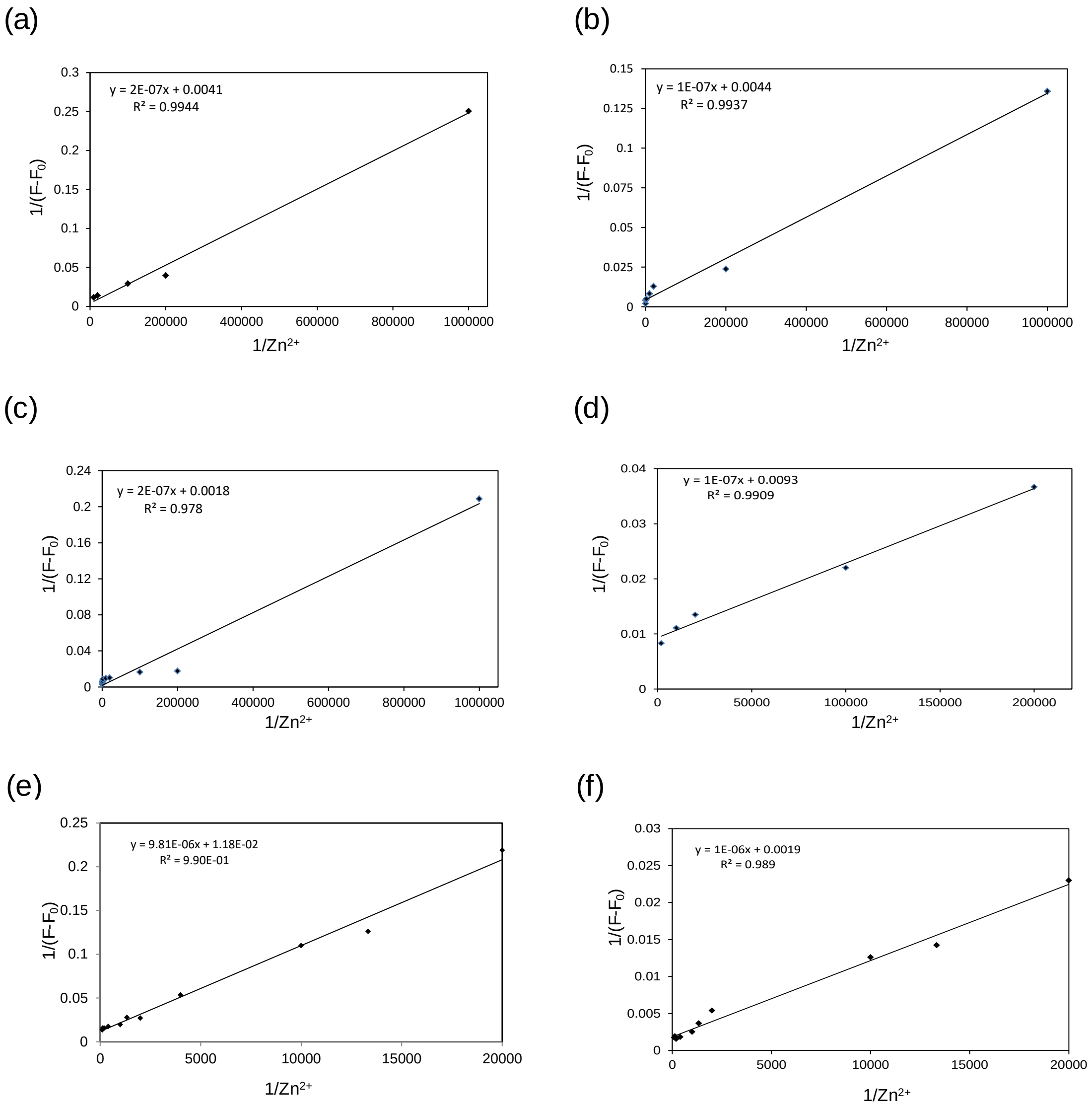

Figure S2. Typical Benesi-Hildebrand analysis of (a) **3a**, (b) **3b**, (c) **4a**, (d) **4b**, (e) **5a** and (f) **5b**.

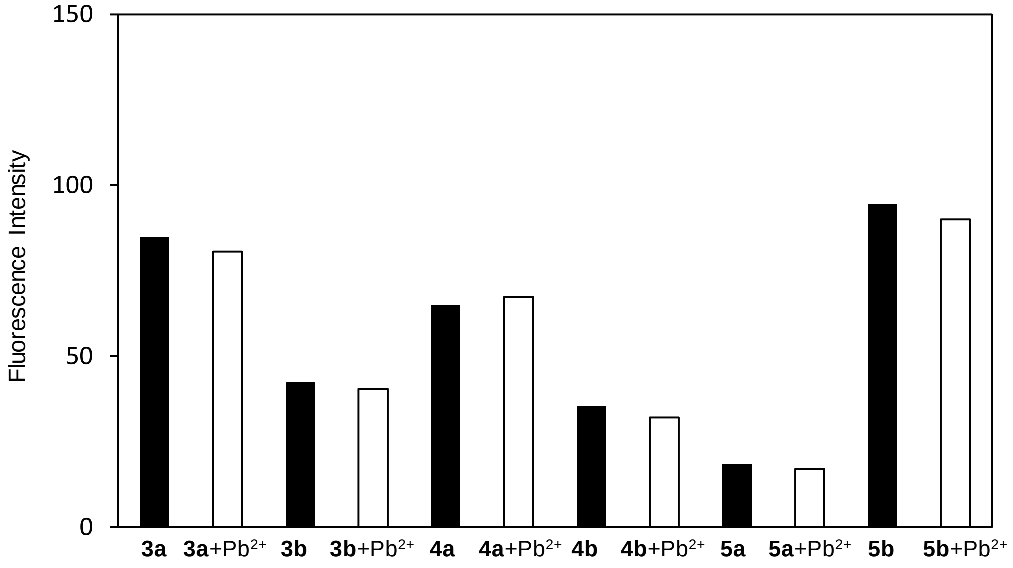

Figure S3. Fluorescence response of (a) **3a** (10^{-5} M, λ_{em} = 381 nm); (b) **3b** (10^{-5} M, λ_{em} = 367 nm); (c) **4a** (10^{-5} M, λ_{em} = 353 nm); (d) **4b** (10^{-5} M, λ_{em} = 360 nm); (e) **5a** (10^{-5} M, λ_{em} = 377 nm); (f) **5b** (10^{-5} M, λ_{em} = 395 nm) upon addition of Pb^{2+} in HEPES buffer (100 mM, 5% DMSO, pH = 7.4). λ_{em} = emission wavelength.
